# Involvement of INF-γ functional single nucleotide polymorphism +874 T/A (rs2430561) in breast cancer risk

**DOI:** 10.1016/j.sjbs.2021.06.083

**Published:** 2021-07-02

**Authors:** Hanan E Al-Rashidi, Sherif Refaat, Enas Ahmed, Dalia T Hussein, Fatma M Eltantawy, Sahar Hamed

**Affiliations:** a Medical Laboratory Technology Department, College of Applied Medical Science, Taibah University, Madinah, Saudi Arabia; b Oncology Center, Mansoura University, Egypt; c Emergency Hospital, Mansoura University, Egypt; d Children's Hospital, Mansoura University, Egypt; e Urology and Nephrology Center, Mansoura University, Egypt

**Keywords:** Breast cancer, INF-γ, Polymorphism, Genotypes, Risk factor, BC, Breast cancer, ICB, inflamed cells of breast, C, controls, ARMS-PCR, amplification refractory mutation system, polymerase chain reaction method, OR, odds ratio, CI, 95% confidence intervals, HER2, human epidermal growth factor receptor 2, ER, estrogen receptor, PR, progesterone receptor, PAM50, Prediction Analysis of Microarray 50, INF-γ, Interferon-γ, TNF-α, tumor necrosis factor-α, TGF-β, transforming growth factor-β, IL, interleukin, SNPs, single nucleotide polymorphism, ISGs, INF-stimulated genes, NK, natural killer cells, Th1, T helper1, CD, cluster of differentiation, IRB, Institutional Review Board, NPI, the mandatory prognostic index, GPI, good prognostic index, MPI, moderate prognostic index, PPI, poor prognostic index, TNBC, Triple Negative BC

## Abstract

According Global Cancer Statistics 2020 GLOBOCAN estimates female breast cancer was found as the most commonly diagnosed cancer, with an estimated 2.3 million new cases (11.7%), and the fourth leading cause (6.9%) of cancer death among women worldwide. Identification of new diagnostic marker sharply characterize the tumor feature is intensive need. The present work was performed to investigate the involvement of the INF-γ + 874 T/A gene polymorphism in different breast cancer prognostic factors. Polymorphism detection analysis was performed on 163 subjects from breast cancer patients, 79 with inflamed cells of breast patients and 144 controls. The gene polymorphism was detected using the amplification refractory mutation system- polymerase chain reaction method (ARMS-PCR). The distribution of INF-γ T + 874A gene polymorphism shows strong significant association between INF-γ + 874 T/A genotypes TT in BC patients (ORTT: 6.41 [95% CI = 2.72–15.1] P < 0.0001) as well as strong significant association regarding T allele (ORT: 1.99 [95% CI = 1.43–2.76] P < 0.0001) when compared to the healthy control. In ICB group the strong association was noted with INF-γ + 874 T/A genotypes AT genotype (ORAT: 2.28 [95% CI = 1.22–4.29] P = 0.007). From the different histological BC hormonal markers the human epidermal growth factor receptor 2 (HER2) was showing significant association in INF-γ + 874 T/A genotypes TT (P = 0.03) and recessive model (TT versus AA + AT P = 0.03). Concerning different BC prognostic models, the poor prognostic one of luminal B, (ER^+ve^ PR^+ve^ Her2^+ve^) show significant association in the host INF-γ + 874 T/A genotype (TT, P = 0.03) and recessive model (TT versus AA + AT P = 0.02) when compared to the good prognostic hormonal status luminal A model, (ER^+ve^ PR^+ve^ Her2-ve). It seems that this is the first study that interested in correlate the INF-γ + 874 T/A gene polymorphisms in Egyptian BC patients. T allele, TT genotype and recessive model of the INF-γ + 874 T/A gene variants were documented as risk factors for BC pathogenesis. It may be used as practical biomarker to guide the BC carcinogenesis and risk process.

## 1 Introduction:

Breast cancer (BC), worldwide is showing persistent diagnosing type of cancer which growing by about more than two million new cases each year presented over (11.7%) of all diagnosed cancer, and found to be the primarily cause of women death which accounts for (6.9%) of total cancer deaths. The death rate in female BC was found more in transitioning countries when compared to transitioned countries (15.0 to 12.8 per 100,000 cases), **(**Sung et al., 2021**).** In Egypt, breast cancer represents the highest incidence female's cancer types; with more than (32%) and a three-fold increase is predicted by 2050 as recently reported by the National Cancer Institute (NCI), Egypt **(**NCI, 2020**).** Comparing to USA and other Western societies, Egypt show lower incidence in BC, while Egyptian BC patients shows higher mortality rate. BC is the second-leading cause of death from cancer in Egyptian women. Egyptian BC patients with no family history of BC shows 85% of all diagnosed BC. This may explained by the genetic mutations that happen as a result of the aging or life style with a tendency to occur in younger age groups with advanced stages **(**Feng et al., 2018, National Cancer Registry Program of Egypt, Saleh et al., 2021**).**

Different classical pathological markers was used to conform patients clinical character like tumor size, as well as estrogen receptor (ER), progesterone receptor (PR) and human epidermal growth factor receptor 2 (HER2) statuses. The high‐risk patients should be identified at the earliest stage by applying a novel diagnostic and therapeutic regimen. The Prediction Analysis of Microarray 50 (PAM50) recently clinically classifies breast cancers according the level of gene expression into five subgroups. This classification totally summarizes the classical pathological markers involved in BC. For example, affluent infiltration by lymphocytes was found to be high in ER negative more than ER positive BC. Regarding chemotherapy, increased response to neo-adjuvant and adjuvant treatment with high immune infiltration has been documented **(**Pruneri et al., 2018, Dannenfelser et al., 2017**).** The immune infiltration in BC was detected using different methods but most with invasive application. Little studies have assessed cytokine serum levels as marker for infiltration of the immune cells **(**Jabeen et al., 2019, Espinoza et al., 2016, Curtis et al., 2012**).**

It is well known that the immune system has an important role in cancer recognition and eradication **(**Blankenstein et al., 2012**).** Local immune response is induced within cancers by modifying the microenvironment and/or neo-antigens expression. Describing the behavior of different molecules orchestrating the tumor local immune response can help knowing the effect of these molecules involving the clinical outcome or the tumor progression **(**Alspach et al., 2019**).** These small molecules may secrete by both tumor cells and stromal or immune cells. As well as, they have the ability to orchestrate immune response regulation. These molecules including different members like, cytokines, growth factors, and chemokines. Cancer detection or tumor pathogenesis can be monitoring by evaluating serum levels of cytokines as interesting noninvasive biomarker **(**Jabeen et al., 2019, Kaur et al., 2018**).** The altered levels of pro- and anti-inflammatory cytokines in cancer document the strong association between inflammation and cancer with high cytokine level related to bad tumor prognosis associated to aggressive cancer and advanced stages. Interferon-γ (INF-γ), tumor necrosis factor-α (TNF-α), transforming growth factor-β (TGF-β), and interleukin (IL)-1, −2, −6, −10, −17 are predominant present in tumor microenvironment and represent different type of these cytokines. These cytokines promote tumorigenesis using different signaling pathways including metabolism, angiogenesis, proliferation, apoptosis and metastasis **(**Kaur et al., 2018, Landskron et al., 2014**).** Changes in cytokine level profile in the tumor were found to be linked to cytokine gene polymorphism which represents a functional single nucleotide polymorphism (SNPs). Cytokines and their receptors SNPs may occur in coding region (not common) or non-coding (common, as in promoters and introns) regions **(**Dondeti et al., 2016**).**

Interferon (INF) family consist of three types of INFs, all are induce INF-stimulated genes (ISGs) during the course of immune response. They are, type I (INF-α/β), type II (INF-γ) and type III (INF-λ, IL-28A/B and IL-29) (Aqbi et al., 2018). From the INF family INF-γ is the only type which can orchestrate both innate and adaptive immune responses against viruses, bacteria, and tumors. This role was found to be contradictory which affect the pathologic inflammatory processes initiating or preventing the disease. INF-γ way within the tumor microenvironment was found to affect the fate of tumor in a cancer bearing individual **(**Alspach et al., 2019**).** INF-γ may be produced in perverse manner which associated with disease pathology, even in chronic autoimmune diseases. INF-γ producing cells are restricted to T lymphocytes and natural killer (NK) cells representing innate immune responses. Within the adaptive immune response, T helper1 (Th1) CD4 + T cells and CD8 + cytotoxic T cells are coexisting in the polarized immune response **(**Mah and Cooper, 2016, Negishi et al., 2017**).** First INF-γ was known exclusively as antitumor, that it has anti-proliferative effects on tumor cells by different actions like, promote myeloid cell activation, antigen presenting cells activation and induce directed cellular migration. Indirectly, it can produce a variety of chemokines, and encourage CD4 + T-cell polarization to Th1 and maturation of CD8 + T cells into cytotoxic T cells. In tumor cells and host cells, INF-γ was found to induce paradoxes responses that facilitate tumor outgrowth leading to initiation of immunosuppressive tumor microenvironment and adaptive resistance which result in tumor-specific T cells suppression **(**Aqbi et al., 2018, Mojic et al., 2018**).**

In advanced stage of breast cancer continues stimulation and suppression of the immune response may lead to confused cytokines production. While breast cancer tumors are showing a highly diversified pattern, in patients with more advanced tumor phenotype it showing concerted clinical characteristics. In breast cancer, the levels of cytokine in serum may be correlated to specific immune cell types at the tumor microenvironment which experienced the important of measuring serum cytokine levels **(**Jabeen et al., 2019, Kaur et al., 2018, Yaghoobi et al., 2018**).** The production of INF is correlated to the functional SNPs INF-γ T + 874A, presenting the T allele contributing to the high INF production, and the A allele responsible for the low INF production. This gene polymorphism is located at the position + 874 of the intron 1 of INF-γ gene (T + 874A INF-γ). Even in healthy subjects carriers the T allele, levels of INF cytokines are higher comparing to those with the A allele carriers. Now it is clearly known that the T to A polymorphism can directly affect the level of INF production **(**Pravica et al., 2000, Karimi et al., 2012**).**

The effect of different cytokines gene polymorphisms in breast cancer have been documented in several studies confirming its association to BC involvement **(**Kober et al., 2016, Alexander et al., 2018, Jung et al., 2021**)**. While, the association of INF-γ gene polymorphism to breast cancer susceptibility, prognosis and tumor characteristic features are still debate. The controversial role of its SNPs with BC remains continued **(**Ge et al., 2014, Li et al., 2014, Szkup et al., 2019, Cameron et al., 2021**).** The aim of this work was to investigate in depth the involvement of the INF-γ + 874 T/A gene polymorphism in the pathogenesis and different breast cancer characteristic as well as the prognostic factors.

## 2 Materials and Methods:

### 2.1 Ethical declaration:

The patients were admitted to Mansoura University Oncology Center Hospitals, Mansoura, Egypt, over the years 2019 and 2020. The protocol approval was allowed by the Institutional Review Board (IRB) at Mansoura University before starting the study. All methods were performed in accordance to the guidelines and regulations proposed in the 1975 Declaration of Helsinki. Informed consent letter was obtained from all the participants. All the patient related data including biological samples were anonymized to ensure confidentiality.

### 2.2 Patients and controls:

BC female patients 163 the median age = 52.7 years, (age range = 27– 80 years). For each patient, tumor size, as well as estrogen receptor (ER), progesterone receptor (PR) and human epidermal growth factor receptor 2 (HER2) statuses were detected by which the BC group was further correlate these separate individual prognostic factors to the INF-γ + 874 T/A genotypes. BC patients group have been recently diagnosed as having breast cancer with no chemo/radiotherapy involvements. NPI, the mandatory prognostic index accurately predicts survival in BC patients **(**Todd et al., 1987**)** was calculated for each BC patient. Three prognostic groups were cut-off points separated. They were (NPI of < 3.4) represent the good prognostic index (GPI), (NPI of 3.41–5.4) was performed as the moderate prognostic index (MPI) and finally the (NPI of > 5.41) were illustrating the poor prognostic index (PPI). The equation used in NPI quantitation is:

NPI= (0.2 X tumor size) + Node status + Grade status.

Another two groups were recruited, inflamed cells of breast group (ICB) of 79 patients and 144 volunteer of control group (C) were recruited as cancer-free and donors of solid organ from Mansoura University with median age of 45.9 years, (age range 36– 63 years).

### 2.3 DNA extraction and INF-γ gene + 874 T/A (rs2430561) genotyping:

EDTA containing tubes were used to collect blood samples. DNA was extracted from puffy coats of EDTA samples were it can be collected after spin at 2500 g for 9 min at RT. DNA extraction was performed according to the commercial kit procedure Promega DNA extraction kit (Promega. USA. A1120).

**Fig. 1:**
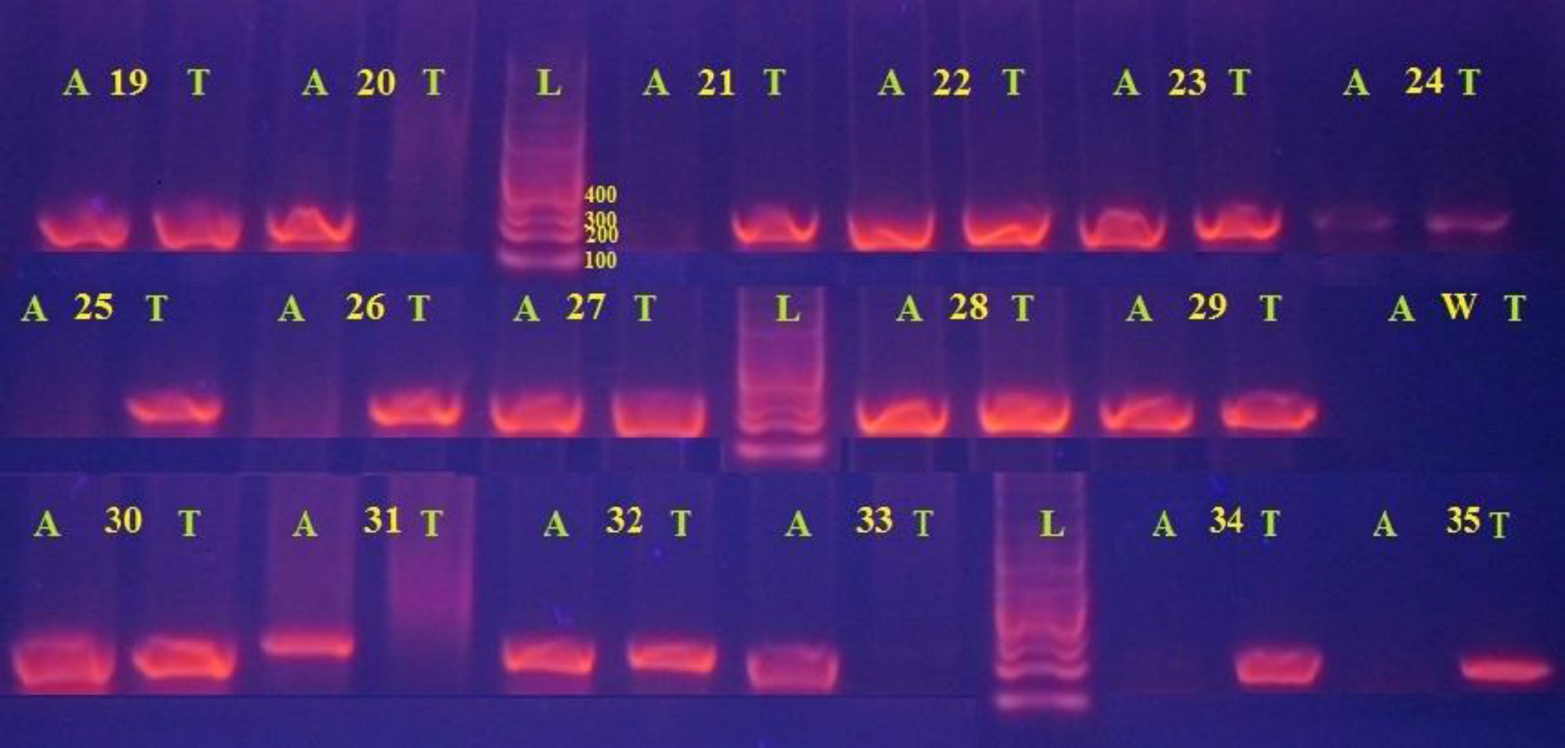
Agarose gel electrophoresis of the INF-γ (+874 T/A) different genotypes. The band of each A or T allele was detected at 263 bp. Lane L indicate DNA Ladder 100 bp marker (v*i*vant*i*s, Malaysia), each genotype represented with two lanes, one for A allele and the other one is for T allele. AA genotype indicated in sample # 20, 31 and 33 where A allele bands are the only present. TT genotype indicated in sample # 21, 25, 26, 34 and 35 where T allele band is the only present and AT genotype indicated in the rest of the samples where both allele bands are present. Negative control water (w) from each allele represents no band in each allele.

The INF-γ + 874 T/A genotypes were determined using the amplification refractory mutation system- polymerase chain reaction method (ARMS-PCR) according to **(**Karimi et al. 2012**).** Amplification of genomic DNA for polymorphic analysis was performed using a Gene Amp PCR system (Thermo Scientific ARKTIK thermal cycler). ARMS-PCR use three different primers, one forward for A-allele; the other forward primer represent T-allele and finally the common reverse primer, they all prepared by (Eurofins, genomics, Germany). INF- γ + 874 T/A genotyping was performed in two separate PCR reaction mixtures of 20 μL for each allele. The mixture was containing 10 μL of 2X ViRed Taq Master Mix (v*i*vant*i*s, Malaysia), 2 μL of each primer (10 pmol/ml), and 2 μL of nuclease free water and 4 μL represent 200 ng of genomic DNA. Thermal condition was as follows: 5 min at 94 °C followed by 10 cycles of 60 sec at 94 °C, 60 sec at 62 °C, 60 sec at 72 °C, and 25 cycles of 60 sec at 94 °C, 60 sec at 58 °C, and 60 sec at 72 °C, with a final extension for 7 min at 72 °C. The amplified PCR products were separated at 263 bp were electrophoresed on a 1.5% agarose gel staining with ViSafe Red Gel Stain (v*i*vant*i*s, Malaysia). The presence of an allele-specific band of the expected size (263 bp) in a lane represented as evidence for the allele presence, whereas the absence of an allele-specific band indicated the absence of that allele **(**Fig. 1**).** The test consistency and reproducibility were confirmed by randomly selecting 15% of the DNA samples to repeat the PCR for second time and where no misapprehension in the genotyping. The results were totally concordant with the former ones.

INF- γ T + 874A primers:

Common: 5′-TCAACAAAGCTGATACTCCA-3′

A allele: 5′-TTCTTACAACACAAAATCAAATCA-3′

T allele: 5′-TTCTTACAACACAAAATCAAATCT-3′

### 2.4 Statistics:

The gene counting method was used to calculate allelic frequencies in all subjects participating in the study. The genotypes of INF-γ + 874 T/A and allele frequencies in BC patients were compared to ICB and controls using chi-square test. Relative risk for disease were calculated by Odds ratios (OR) and 95% confidence intervals (CI). The correlation values of histological and clinical data with the INF-γ + 874 T/A genotypes in BC patients were calculated using the same tests. NPI quantitatively compared to the genotypes of INF-γ + 874 T/A using two-tailed Student’s *t*-test. Statistical significance was assumed at the P < 0.05 level. The SPSS statistical software package version 21.0 for Windows (Chicago, Illiniois, USA) was used for the statistical analysis.

## 3 Results

### 3.1 Distribution of INF-γ + 874 T/A (rs2430561) genotype in different studied groups

A total of 247 female breast patients were participated in the study in addition to 144 healthy unrelated individuals from the same locality. The amplified PCR product for INF-γ + 874 were detected at 263 base pair as shown in **(**Fig. 1**)**, based on these results, in different studied groups, the genotypes and the alleles of the INF-γ + 874 T/A genes polymorphism were determined and evaluated in comparison with their respective healthy controls. Results show in **(**Table 1**)**, pointed out the different genetic models which revealed in the host INF-γ + 874 (TT + AT genotypes) versus those with the AA genotype (dominant model) revealed strong significant association with BC group (P < 0.0001) and significant association with ICB group (P = 0.01) total number of cases in comparison with their respective controls and no significant association between BC and ICB groups (P = 0.43), [OR (95% CI) were 2.6 (1.57–4.3), 2.15 (1.15–4.02) and 0.83 (0.43–1.58), respectively].

**Table 1:** Distribution of different genotype of INF-γ + 874 T/A (rs2430561) with risk estimate and allele frequencies in control, BC and IBC groups in different genetic models.

**IFN Genotype**	**Group's # (%)**			
	**Control 144**	**BC 163**	**IBC 79**	
**AA**	56 (38.9)	32 (19.6)	18 (22.8)	
**AT**	79 (54.9)	98 (60.1)	58 (73.4)	
**TT**	9 (6.2)	33 (20.3)	3 (3.8)	
**Allele**				
**A**	191 (66.3)	162 (49.7)	94 (59.5)	
**T**	97 (33.7)	164 (50.3)	64 (40.5)	
**Statistics**	**TT + AT vs AA (Dominant)**			**BC vs IBC**
**OR**		2.6	2.15	0.83
**95% CI**		(1.57–4.3)	(1.15–4.02)	(0.43–1.58)
**Sig. (P)**		0.0001	0.01	0.34
	**TT vs AA + AT (Recessive)**			**BC vs IBC**
**OR**		0.26	1.7	6.43
**95% CI**		(0.12–0.57)	(0.45–6.48)	(1.9–21.7)
**Sig. (P)**		0.0001	0.32	0.0001
	**AT vs AA + TT (Overdominant)**			**BC vs IBC**
**OR**		0.8	0.44	0.55
**95% CI**		(0.51–1.27)	(0.24–0.8)	(0.3–0.98)
**Sig. (P)**		0.2	0.005	0.029
	**T allele vs A allele**			**BC vs IBC**
**OR**		1.99	1.34	0.67
**95% CI**		(1.43–2.76)	(0.9–2)	(0.46–0.99)
**Sig. (P)**		0.0001	0.09	0.027
	**AA vs AT (Codominant)**			**BC vs IBC**
**OR**		2.17	2.28	1
**95% CI**		(1.28–3.67)	(1.22–4.29)	(0.54–2)
**Sig. (P)**		0.003	0.007	0.51
	**AA vs TT**			**BC vs IBC**
**OR**		6.41	1.04	0.16
**95% CI**		(2.72–15.1)	(0.25–4.25)	(0.04–0.6)
**Sig. (P)**		0.0001	0.6	0.003

Similarly, host INF-γ + 874 (TT versus AA + AT genotypes) (recessive model) revealed high significant association with BC group (P < 0.0001) and no significant association with ICB group (P = 0.32) total number of cases in comparison with their respective controls and high significant association between BC and ICB groups (P < 0.0001), [OR (95% CI) were 0.26 (0.12–0.57), 1.7 (0.45–6.48) and 6.43 (1.9–21.7), respectively]. In the other hand allelic frequency of T versus A revealed a significant association with BC group (P < 0.0001) and no significant association with ICB group (P = 0.09) total number of cases in comparison with their respective controls and significant association between BC and ICB groups (P = 0.027), [OR (95% CI) were 1.99 (1.43–2.76), 1.34 (0.9–2) and 0.67 (0.46–0.99), respectively].

Testing for the codominant model (AA vs. AT) revealed that there was a significant difference between healthy controls and patients groups (P = 0.003 in BC, and P = 0.007 in ICB) while the codominant model (AA vs. TT) revealed that there was a high significant difference between healthy controls and BC group only (P < 0.0001) and no differences ICB group (P = 0.6). The complete details of INF-γ + 874 A/T polymorphism for all studied subjects are summarized in **(**Table 1**).** These results support a potential role of INF-γ + 874 T/A in the pathogenesis of breast cancer among the studied Egyptian population.

### 3.2 Distribution of INF-γ + 874 T/A (rs2430561) genotype in different variant of BC Group

Demographic, clinico-pathological data and biomarker parameters of the study participants which have been gathered from patients’ medical records were shown in **(**Table 2**, First Column)**. Different features listed in the table represent the number and percentage of each variant in relation to the BC group, among these features the predominant cancer stage was stage II (67.5%), node status was N0 (35.6%), cancer grade was grade II (71.1%), tumor size was ≥ 2 cm- 5 cm (56.5%), NPI was > 3.4–5.4 (74.3%), positive ER was (79.8%), positive PR was (75.5%), negative Her2/neu expression was (55.2%), negative metastasis was (85.3%) and left operated breast was (61.3%). Different INF-γ + 874 T/A (rs2430561) genotypes in BC group was 32 (19.6%), 98 (60.1%) and 33 (20.3%) for AA, AT and TT genotype respectively.

**Table 2:** Characteristic frequency of tumor characters in breast cancer patients (163 Patients, first column). Distribution of different genotype of INF-γ + 874 T/A (rs2430561) gene in different tumor characters.

**Variables**	**Patient number (percentage)**		
**Genotype**	**AA**	**AT**	**TT**
**(163 Patients)**	**32 (19.6)**	**98 (60.1)**	**33 (20.3)**
**Cancer stage**			
**26 (15.9) T1**	2 (7.7)	19 (73)	5 (19.3)
**110 (67.5) T2**	25 (22.7)	62 (56.4)	23 (20.9)
**21 (12.9) T3**	5 (23.8)	12 (57.2)	4 (19)
**6 (3.7) T4**	0 (0)	5 (83.3)	1 (16.7)
**Node Status**			
**58 (35.6) N0**	9 (15.6)	36 (62)	13 (22.4)
**40 (24.5) N1**	6 (15)	27 (67.5)	7 (17.5)
**40 (24.5) N2**	10 (25)	19 (47.5)	11 (27.5)
**25 (15.4) N3**	7 (28)	16 (64)	2 (8)
**Overall grade**			
**3 (1.8) G1**	0 (0)	3 (1 0 0)	0 (0)
**116 (71.2) G2**	24 (20.7)	69 (59.5)	23 (19.8)
**44 (27) G3**	8 (18.2)	26 (59.1)	10 (22.7)
**Tumor size**			
**26 (15.9) < 2 cm**	2 (7.7)	18 (69.2)	6 (23.1)
**92 (56.5) 2**–**5 cm**	20 (21.7)	51 (55.5)	21 (22.8)
**45 (27.6) > 5 cm**	10 (22.2)	29 (64.4)	6 (13.4)
**NPI**			
**9 (5.5) > 2.4**–**3.4**	2 (22.2)	6 (66.7)	1 (11.1)
**121 (74.2) > 3.4**–**5.4**	21 (17.3)	75 (62)	25 (20.7)
**33 (20.3) > 5.4**	9 (27.3)	17 (51.5)	7 (21.2)
**Estrogen receptor**			
**33 (20.3) Negative**	4 (12.1)	22 (66.7)	7 (21.2)
**130 (79.7) Positive**	28 (21.5)	76 (58.5)	26 (20)
**Progesterone receptor**			
**40 (24.5) Negative**	5 (12.5)	26 (65)	9 (22.5)
**123 (75.5) Positive**	27 (22)	72 (58.5)	24 (19.5)
**Her2/neu expression**			
**90 (55.2) Negative**	21 (23.3)	56 (62.2)	13 (14.5)
**73 (44.8) Positive**	11 (15)	42 (57.5)	20 (27.5)
**Metastasis**			
**139 (85.3) Negative**	29 (20.8)	86 (61.8)	24 (17.4)
**24 (14.7) Positive**	4 (16.7)	12 (50)	8 (33.3)
**Operation Type**			
**100 (61.4) Lt MRM**	18 (18)	58 (58)	24 (24)
**63 (38.6) Rt MRM**	14 (22.2)	40 (63.5)	9 (14.3)

The distribution of different genotype of INF-γ + 874 T/A (rs2430561) gene in different variables of tumor in breast cancer patients (163 Patients) were detailed in **(**Table 2**).** among the predominant of these features AT genotype show the most prevalence genotype in cancer stage was in stage T4 (83.3%), in node status was N1 (67.5%), in cancer grade was grade I (100%), in tumor size was (≥2 cm- 5 cm) (69.2%), in NPI was (>2.4–3.4, GP) (66.7%), in negative ER was (66.7%), in negative PR was (65%), in negative Her2/neu expression was (62.2%), in negative metastasis was (61.8%) and right operated breast was (63.5%). The AA and TT genotypes show mostly the same presentation in different BC characteristic variables of the tumor. TT genotype tends to be predominant in the worse variable of T4, G3, negative ER and PR, positive Her2new and metastasis. AA genotype tends to be predominant in the worse variable of N3, tumor size and NPI. Detailed distribution of different genotype of INF-γ + 874 T/A (rs2430561) gene in different variables of tumor in breast cancer patients (163 Patients) were presented in **(Supplement Tables,** (ER) **1**, (PR) **2**, (HER2) **3** and **(**Metastasis) **4**).

By comparing the different models of INF-γ + 874 T/A (rs2430561) genotype as a risk estimate with different variables of tumor in BC group, results revealed no association with ER, PR, metastasis or operation type **(Supplement Tables,** (ER) **5,** (PR) **6,** (Metastasis) **7** and (Operation Type) **8)**. While a significant association in the host INF-γ + 874 T/A genotype with **Her2/neu** expression marker, in the codominant model (AA vs. TT, P = 0.03) as well as recessive model (TT versus AA + AT P = 0.03) with the positive Her2/neu expression marker, (Table 3**).**

**Table 3:** Distribution of different genotype of INF-γ + 874 T/A (rs2430561) with risk estimate in response to Her2/neu expression marker in BC group.

**Model**	**Genotype # (%)**		**OR (95% CI)**	**Sig. (P)**
**Codominant**	**Negative 90 (55.2)**	**Positive 73 (44.8)**		
**AA**	21 (23.3)	11 (15)	1	
**AT**	56 (62.2)	42 (57.5)	1.43 (0.62–3.29)	0.26
**TT**	13 (14.5)	20 (27.5)	2.94 (1.07–8.06)	0.030
**Dominant**	AA vs AT + TT		1.71 (0.76–3.84)	0.13
**Recessive**	AA + AT vs TT		0.45 (0.2–0.98)	0.032
**Overdominant**	AA + TT vs AT		1.21 (0.65–2.28)	0.32

When testing the host INF-γ + 874 T/A genotype in different BC prognostic models **(**Salimifard et al., 2020**)** the very poor prognostic model **(Triple –ve model)** which show negative expression for different hormonal status (11 cases) as well as the poor prognostic hormonal status **Her2 enriched model (ER^-ve^ PR^-ve^ Her2^+ve^)** model (14 cases), we found no statistical significant differences within different host INF-γ + 874 T/A genotype when compared to the good prognostic model (64 cases) hormonal status **luminal A model, (ER^+ve^ PR^+ve^ Her2^-ve^)**, **(Supplement Tables (**Triple –ve model**) 9, (**Her2 enriched model**) 10 and (**Triple –ve model vs Her2 enriched model**) 11).** While a significant association in the host INF-γ + 874 T/A genotype with other poor prognostic model of **luminal B model, (ER^+ve^ PR^+ve^ Her2^+ve^)** of hormonal status (56 cases), in the codominant model (AA vs. TT, P = 0.03) as well as recessive model (TT versus AA + AT P = 0.02) when compared to the good prognostic hormonal status **luminal A model, (ER^+ve^ PR^+ve^ Her2^-ve^)**, **(**Table 4**).**

**Table 4:** Distribution of different genotype of INF-γ + 874 T/A (rs2430561) with risk estimate in the poor prognosis luminal B model (ER^+ve^ PR^+ve^ Her2^+ve^) of hormonal status vs good prognostic hormonal status luminal A model (ER^+ve^ PR^+ve^ Her2^-ve^) in BC group.

**Model**	**Genotype # (%)**		**OR (95% CI)**	**Sig. (P)**
**Codominant**	**Good prognostic 64 cases**	**Poor prognostic 56 cases**		
**AA**	17 (26.6)	10 (17.8)	1	
**AT**	39 (60.9)	30 (53.5)	1.31 (0.52–3.26)	0.36
**TT**	8 (12.5)	16 (28.7)	3.4 (1.07–10.77)	0.03
**Dominant**	AA vs AT + TT		1.66 (0.69–4.01)	0.17
**Recessive**	AA + AT vs TT		0.53 (0.14–0.91)	0.02
**Overdominant**	AA + TT vs AT		1.35 (0.65–2.79)	0.26

### 3.3 Distribution of INF-γ + 874 T/A (rs2430561) genotype according NPI in BC Group

Regarding NPI, the frequency among different INF-γ + 874 T/A (rs2430561) genotype was listed in (**Supplement Table 12).** No significant differences have been noted within different genotypes when using student t- Test. The different NPI were (4.82 ± 0.86 for AA, 4.65 ± 0.78 for AT and 4. 69 ± 0.79 for TT) respectively and the significant were (P = 0.29 for AA vs AT, P = 0.51 for AA vs TT and P = 0.29 for AT vs TT) respectively. Similarly, when different genotypes were tested within different NPI groups GPI, MPI and PPI **(**Table 5**),** no significant differences were observed between different INF-γ + 874 T/A (rs2430561) genotypes. Detailed significance was summarized in **(Supplement Table 13)** for different hormonal status. Similarly, no significant differences in NPI when different INF-γ + 874 T/A (rs2430561) genotype where tested in response to different hormonal markers, detailed significance were summarized in **(Supplement Tables** (ER) **14**, (PR) **15** and (HER2) **16).**

**Table 5:** Means and standard error of the mean of NPI for different genotype of INF-γ + 874 T/A (rs2430561) within different prognostic groups in BC group.

**Group**	**IFN**	**N**	**Mean**	**Std. Deviation**	**Std. Error**	**Sig.^a^**
**GPI**	**AA**	2	3.390	0.0141	0.0100	
	**AT**	6	3.190	0.2054	0.0839	0.063
	**TT**	1	3.400	0.0	0.063	0.6670.387^b^
**MPI**	**AA**	21	4.511	0.5765	0.1258	
	**AT**	75	4.484	0.4768	0.0551	0.843
	**TT**	25	4.448	0.6145	0.843	0.7200.762^b^
**PPI**	**AA**	9	5.878	0.2108	0.0703	
	**AT**	17	5.918	0.2675	0.0649	0.681
	**TT**	7	5.743	0.1813	0.681	0.1910.082^b^

a = significance of AA genotype vs other genotype, b = significance of AT genotype vs TT genotype., GPI = Good Prognosis., MPI = Moderate Prognosis., PPI = Poor Prognosis

### 3.4 Distribution of INF-γ + 874 T/A (rs2430561) genotype according metastasis in BC Group

Metastasis the most worth complication in BC was detected in 24 patients, where 4 patients show INF-γ + 874 T/A (rs2430561) AA genotype, 12 patient show AT genotype and 8 represented with TT genotype. The most presented metastasis was in bone metastasis presented in 9 cases, bone and LN in 5 cases, lung in 5 cases, bone and liver in 3 cases, bone and lung in one case and another case the metastasis goes to brain, bone and LN. Detailed presentation of different INF-γ + 874 T/A (rs2430561) genotype showing metastasis was presented in (Table 6**).** When concerning different predictive models, the reference one with good prognosis **luminal A model, (ER^+ve^ PR^+ve^ Her2^-ve^)** model we found (7/64 metastasis cases, 11%) one case in AA genotype of INF-γ + 874 T/A (rs2430561) SNP with bone metastasis, 5 cases in AT genotype (2 with lung and 3 with bone) metastasis and one cases in TT genotype (bone & lung metastasis). The very poor prognostic **(Triple –ve model)** show (2/11 metastasis cases, 18%) all are in TT genotype (one with bone & liver and one with lung metastasis). The poor prognostic hormonal status **Her2 enriched model (ER^-ve^ PR^-ve^ Her2^+ve^)** model shows (1/14 case 7%, with lung metastasis in TT genotype). The other poor prognostic model of **luminal B model, (ER^+ve^ PR^+ve^ Her2^+ve^)** of hormonal status show (9/ 56 metastasis cases, 16%), we found one case in AA genotype with bone, brain & LN metastasis, 5 cases in AT genotype (one with lung and 4 with bone & LN) metastasis and 3 cases in TT genotype (one with bone & LN and 2 with bone & liver).

**Table 6:** Distribution of different metastasis sites in different INF-γ + 874 T/A (rs2430561) genotype.

**Site of Metastasis**	**AA**	**AT**	**TT**
**Bone**	3	4	1
**Bone & LN**	0	4	1
**Bone & Liver**	0	0	3
**Bone & Lung**	0	1	1
**Bone & Brain & LN**	1	0	0
**Lung**	0	3	2

## 4 Discussion

Breast cancer is known as complex and multifactorial disease, the interaction between environmental and genetic factors probably important in initiation and development of the disease **(**Sung et al., 2021**).** As cytokines are known by molecules regulating the immune response, they are still poorly characterized in breast cancer. High risk of breast cancer susceptibility may contribute to different identified genetic loci **(**Bouchardy et al., 2013, Nickels et al., 2013**).** The increasing guided facts has demonstrated that the levels of certain cytokines production, including interferon-γ (INF-γ), may inhibits the growth of many originating cell lines tumors, as well as may be involved in breast cancer development **(**Li et al., 2014**).**

This study determined the association of interferon-γ functional single nucleotide polymorphism (+874 T/A) in Egyptian patients with breast cancer. It is now well established that breast cancer is the most persistent diagnosed cancer worldwide and considered a chief reason of cancer mortality among females worldwide **(**Sung et al., 2021**).** In Egypt, the dispersal of BC is growing and it remains a major health problem of the country with no solution. It constitutes 33% of female cancer cases and>22,000 new cases diagnosed each year **(**Ibrahim et al., 2014**).** This is expected to rise exponentially over the next years given the enlarging population. A three-fold increase is predicted by 2050 as recently reported by the National Cancer Institute (NCI), Egypt **(**NCI, 2020**).**

Analysis of INF-γ + 874 A/T polymorphism on 163 Egyptian patients with BC, 79 inflamed cells of breast (ICB) and 144 healthy controls from the same area, showed strong significant association of INF-γ + 874 genotype with BC patients with both codominant models (AA vs. AT) and (AA vs. TT), dominant model (TT + AT versus AA), recessive model (TT versus AA + AT) as well as and allelic frequency of INF-γ + 874 (T versus A). Our findings seem not to be reported before, where the INF-γ + 874 TT genotype and T allele were found to be associated with high transcription of INF-γ gene in BC, in which they are involved in the higher production of INF-γ **(**Jabeen et al., 2019, Kaur et al., 2018**).** Different studies were examined the INF-γ + 874 SNPs in BC with different study interest like, metabolic syndrome, fatigue and other symptoms following breast cancer treatment **(**Szkup et al., 2019, Cameron et al., 2021**).** Even they have another interest research; their results support our result confirming the association of INF-γ + 874 TT genotype and T allele in BC patients. These data supports our results either directly or indirectly. Moreover, a *meta*-analysis study confirms our results by figure out a higher frequency of T allele or TT genotype which significantly involved in BC patients among Asians **(**Li et al., 2014**).**

In contrast of these results, a lack of association between INF-γ + 874 T/A gene Polymorphism and cancer risk was reported by Ge and coworkers **(2014)**, this may they include 38 studies with different cancer types and pool all these type results together while each cancer type has different response to INF-γ + 874 T/A gene Polymorphism. The six studies concerning breast cancer their figure of both AA and TT genotype have almost the same number, while other type of cancer may has different figure for example in cervical cancer AA genotype has twice or more higher number compared to TT genotype. So, instead pooling all cancer types in one analysis, each cancer type should be treated as separate identity, this may change the article conclusion **(**Ge et al., 2014**).**

The ICB group was used as another control to test the carcinogenesis in BC patient. Analysis of INF-γ + 874 A/T polymorphism on 70 ICB group revealed same figure like healthy control group where no association to the INF-γ + 874 T/A SNP in codominant model (AA vs. TT), recessive model (TT versus AA + AT) as well as and allelic frequency of INF-γ + 874 (T versus A) when comparing to healthy control group. While, when comparing ICB group to BC group a significant association to the INF-γ + 874 T/A SNP in codominant model (AA vs. TT), recessive model (TT versus AA + AT) as well as and allelic frequency of INF-γ + 874 (T versus A). No existed data to compare our results with, but the results confirm the strong involvement of INF-γ + 874 T/A SNP in BC patients' carcinogenesis and not ICB patients.

Within the BC group, we try to make different correlations between INF-γ + 874 T/A gene Polymorphism and hormonal analysis and Predictive Index (NPI). No association has been noted with INF-γ + 874 T/A gene polymorphism in response to ER or PR hormonal status **or** metastasis **(Supplement Tables, 5**–**7).**, while the human epidermal growth factor receptor 2 (Her2) show a significant association to INF-γ + 874 T/A SNP (P = 0.03) in the codominant model as well as recessive model (AA vs. TT and TT versus AA + AT). This confirms the association of TT genotype with the aggressiveness type of BC. When analyzing different prognostic model a significant association in INF-γ + 874 T/A SNP with the poor prognostic model of **luminal B model, (ER^+ve^ PR^+ve^ Her2^+ve^)** in the codominant model (AA vs. TT, P = 0.03) as well as recessive model (TT versus AA + AT P = 0.02) when compared to the good prognostic hormonal status **luminal A model, (ER^+ve^ PR^+ve^ Her2^-ve^).** This again confirms the association of TT genotype with the aggressiveness type of BC. As immunotherapeutic regimen, INFGR1 was found to elevate the growth and metastasis in triple-negative model of breast cancer (TNBC) through interferon-γ signaling that enhance tumor immunogenicity and immune effector cell accumulation. INFGR1 may be a viable selection to reduce metastatic progression in patients with TNBC, especially those who do not benefit from neoadjuvant chemotherapy **(**Oshi et al., 2020, Singh et al., 2020, Zanker et al., 2020**).**

The NPI frequency among different INF-γ + 874 T/A (rs2430561) genotype show no significant differences. Similarly, when different genotypes were tested within different NPI groups GPI, MPI and PPI**,** no significant differences were observed between different INF-γ + 874 T/A (rs2430561) genotypes. As well as, no significant differences in NPI were noted when different markers have been analyzed.

Metastasis is a complex process that involves tumor spread to distant parts of the body from its original site. The exact initiation process of breast cancer metastasis is unknown. Cytokines recently, was found to be the key players closely related to breast cancer metastasis. This knowledge may help in development of new therapeutic approaches to overcome breast cancer metastasis **(**Liang et al., 2019, Mendez-Garcıa et al., 2019**).** INF-γ provides an applicable strategy for cancer therapy when it incorporated with nanoparticles of α-TOS **(**Wu et al., 2020**)**, which significantly inhibited tumor growth and prevented tumor metastasis. As well as, increase tumor evasion may approve with INFGR1 stabilization which promote CD8 + T cell exhaustion in Triple Negative BC (TNBC). Thus the pro-tumorigenic role of INF-γ signaling unravels along with a putative biomarker that can predict patient response to immunotherapies **(**Singh et al., 2020**).** So**,** the proof-of-principle concept to use the antibodies targeting INFGR1, will improve the clinical outcome for patients with TNBC. The involvement of INF-γ gene polymorphism in BC metastasis was not studied before, our study reveals TT genotype of INF-γ + 874 T/A (rs2430561) has more metastatic cases than AA genotype (8/4 cases) from the 24 cases present metastasis in BC. TT genotype also present the only genotype showing metastasis in the worth prognosis model TNBC and **(ER^-ve^ PR^-ve^ Her2^+ve^)** model, this may be for the low number of cases in these models. While, the other models with good prognostic hormonal status **luminal A model, (ER^+ve^ PR^+ve^ Her2^-ve^)** and poor prognostic model of **luminal B model, (ER^+ve^ PR^+ve^ Her2^+ve^)** of hormonal status can show metastatic distribution along the different INF-γ + 874 T/A (rs2430561) genotypes.

## 5 Conclusion:

It seems that this is the first study that interested in correlate the most functional important gene polymorphisms of INF- γ + 874 A/T in Egyptian BC. The study demonstrated strong association between INF-γ-874 A/T polymorphism to be significantly correlated with the aggressive carcinogenesis of BC, suggesting its role in the pathogenesis of BC especially TT genotype and T allele involvements. This study confirm the association of INF-γ-874 A/T polymorphism TT genotype and T allele with the poor prognostic model as well as the metastatic distribution in BC patient. INF-γ-874 A/T gene polymorphism TT genotype and T allele have strong association to breast cancer carcinogenesis, poor prognosis and metastasis. It may be used as practical biomarker to guide the BC carcinogenesis and risk process.

## 6 Ethical considerations**a

Ethical issues (including plagiarism, data fabrication, double publication) have been completely observed by the authors.

## Declaration of Competing Interest

The authors declare that they have no known competing financial interests or personal relationships that could have appeared to influence the work reported in this paper.
