# Supplementary material for: Involvement of INF-γ functional single nucleotide polymorphism +874 T/A (rs2430561) in breast cancer risk
Source: Saudi J Biol Sci. 2021 Jul 2;28(11):6289–96. doi: 10.1016/j.sjbs.2021.06.083 (PMC8568710; doi:10.1016/j.sjbs.2021.06.083)
Supplement: Supplementary data 1 [file mmc1.docx]

**Table 1: Distribution of different genotype of INF-γ+874 T/A (rs2430561) gene in different variables of tumor in breast cancer patients (163 Patients) in response to Estrogen Receptor in BC group.**

| **Variables** | **AA^-ve^** | **AT^-ve^** | **TT^-ve^** | **AA^+ve^** | **AT^+ve^** | **TT^+ve^** |
| --- | --- | --- | --- | --- | --- | --- |
| **ER** | 4 | 22 | 7 | 28 | 76 | 26 |
| **Cancer stage** |  |  |  |  |  |  |
| **T1** | 1 | 3 | 1 | 1 | 16 | 4 |
| **T2** | 2 | 16 | 4 | 23 | 46 | 19 |
| **T3** | 1 | 2 | 1 | 4 | 10 | 3 |
| **T4** | 0 | 1 | 1 | 0 | 4 | 0 |
| **Node Status** |  |  |  |  |  |  |
| **N0** | 1 | 9 | 2 | 8 | 27 | 11 |
| **N1** | 1 | 3 | 2 | 5 | 24 | 5 |
| **N2** | 1 | 5 | 2 | 9 | 14 | 9 |
| **N3** | 1 | 5 | 1 | 6 | 11 | 1 |
| **Overall grade** |  |  |  |  |  |  |
| **G1** | 0 | 2 | 0 | 0 | 1 | 0 |
| **G2** | 3 | 15 | 5 | 21 | 54 | 18 |
| **G3** | 1 | 5 | 2 | 7 | 21 | 8 |
| **Tumor size** |  |  |  |  |  |  |
| **<2cm** | 1 | 2 | 1 | 1 | 16 | 4 |
| **2- 5cm** | 3 | 12 | 4 | 21 | 48 | 20 |
| **>5 cm** | 0 | 8 | 2 | 6 | 12 | 2 |
| **NPI** |  |  |  |  |  |  |
| **>2.4- 3.4** | 0 | 2 | 1 | 2 | 4 | 0 |
| **>3.4- 5.4** | 3 | 17 | 4 | 22 | 52 | 23 |
| **>5.4** | 1 | 3 | 2 | 4 | 20 | 3 |
| **ER** |  |  |  |  |  |  |
| **Negative** | 4 | 22 | 7 | 0 | 0 | 0 |
| **Positive** | 0 | 0 | 0 | 28 | 76 | 26 |
| **PR** |  |  |  |  |  |  |
| **Negative** | 4 | 16 | 5 | 0 | 10 | 4 |
| **Positive** | 0 | 6 | 2 | 28 | 66 | 22 |
| **Her2/neu** |  |  |  |  |  |  |
| **Negative** | 3 | 10 | 3 | 18 | 47 | 10 |
| **Positive** | 1 | 12 | 4 | 10 | 28 | 16 |
| **Metastasis** |  |  |  |  |  |  |
| **Negative** | 4 | 22 | 4 | 24 | 64 | 21 |
| **Positive** | 0 | 0 | 3 | 4 | 12 | 5 |
| **Oper. Type** |  |  |  |  |  |  |
| **Lt MRM** | 3 | 14 | 5 | 15 | 43 | 18 |
| **Rt MRM** | 1 | 8 | 2 | 13 | 33 | 8 |

**Table 2: Distribution of different genotype of INF-γ+874 T/A (rs2430561) gene in different variables of tumor in breast cancer patients (163 Patients) in response to Progesterone Receptor in BC group.**

| **Variables** | **AA^-ve^** | **AT^-ve^** | **TT^-ve^** | **AA^+ve^** | **AT^+ve^** | **TT^+ve^** |
| --- | --- | --- | --- | --- | --- | --- |
| **PR** | 5 | 26 | 9 | 27 | 72 | 24 |
| **Cancer stage** |  |  |  |  |  |  |
| **T1** | 1 | 2 | 0 | 1 | 17 | 5 |
| **T2** | 3 | 17 | 6 | 22 | 45 | 17 |
| **T3** | 1 | 4 | 2 | 4 | 8 | 2 |
| **T4** | 0 | 3 | 1 | 0 | 2 | 0 |
| **Node Status** |  |  |  |  |  |  |
| **N0** | 2 | 9 | 4 | 7 | 27 | 9 |
| **N1** | 1 | 3 | 1 | 5 | 24 | 6 |
| **N2** | 1 | 6 | 3 | 9 | 13 | 8 |
| **N3** | 1 | 8 | 1 | 6 | 8 | 1 |
| **Overall grade** |  |  |  |  |  |  |
| **G1** | 0 | 2 | 0 | 0 | 1 | 0 |
| **G2** | 4 | 17 | 5 | 20 | 52 | 18 |
| **G3** | 1 | 7 | 4 | 7 | 19 | 6 |
| **Tumor size** |  |  |  |  |  |  |
| **<2cm** | 1 | 1 | 0 | 1 | 7 | 2 |
| **2- 5cm** | 4 | 20 | 7 | 20 | 56 | 20 |
| **>5 cm** | 0 | 5 | 2 | 6 | 9 | 2 |
| **NPI** |  |  |  |  |  |  |
| **>2.4- 3.4** | 2 | 1 | 0 | 1 | 4 | 1 |
| **>3.4- 5.4** | 1 | 19 | 7 | 24 | 47 | 23 |
| **>5.4** | 2 | 6 | 2 | 2 | 21 | 0 |
| **ER** |  |  |  |  |  |  |
| **Negative** | 3 | 16 | 5 | 0 | 6 | 2 |
| **Positive** | 2 | 10 | 4 | 27 | 66 | 22 |
| **PR** |  |  |  |  |  |  |
| **Negative** | 5 | 26 | 9 | 0 | 0 | 0 |
| **Positive** | 0 | 0 | 0 | 27 | 72 | 24 |
| **Her2/neu** |  |  |  |  |  |  |
| **Negative** | 4 | 13 | 4 | 17 | 44 | 9 |
| **Positive** | 1 | 13 | 5 | 10 | 28 | 15 |
| **Metastasis** |  |  |  |  |  |  |
| **Negative** | 5 | 22 | 5 | 23 | 64 | 20 |
| **Positive** | 0 | 4 | 4 | 4 | 8 | 4 |
| **Oper. Type** |  |  |  |  |  |  |
| **Lt MRM** | 4 | 18 | 6 | 14 | 50 | 17 |
| **Rt MRM** | 1 | 8 | 3 | 13 | 22 | 7 |

**Table 3: Distribution of different genotype of INF-γ+874 T/A (rs2430561) gene in different variables of tumor in breast cancer patients (163 Patients) in response to Her2/neu expression in BC group.**

| **Variables** | **AA^-ve^** | **AT^-ve^** | **TT^-ve^** | **AA^+ve^** | **AT^+ve^** | **TT^+ve^** |
| --- | --- | --- | --- | --- | --- | --- |
| **Her2/neu** | 21 | 56 | 13 | 11 | 42 | 20 |
| **Cancer stage** |  |  |  |  |  |  |
| **T1** | 1 | 12 | 2 | 1 | 7 | 3 |
| **T2** | 18 | 36 | 9 | 7 | 26 | 14 |
| **T3** | 2 | 4 | 1 | 3 | 8 | 0 |
| **T4** | 0 | 4 | 1 | 0 | 1 | 3 |
| **Node Status** |  |  |  |  |  |  |
| **N0** | 5 | 25 | 6 | 4 | 11 | 7 |
| **N1** | 5 | 16 | 3 | 1 | 11 | 4 |
| **N2** | 7 | 8 | 3 | 3 | 11 | 8 |
| **N3** | 4 | 7 | 1 | 3 | 9 | 1 |
| **Overall grade** |  |  |  |  |  |  |
| **G1** | 0 | 2 | 0 | 0 | 1 | 0 |
| **G2** | 18 | 43 | 9 | 6 | 26 | 14 |
| **G3** | 3 | 11 | 4 | 5 | 15 | 6 |
| **Tumor size** |  |  |  |  |  |  |
| **<2cm** | 1 | 5 | 1 | 0 | 2 | 1 |
| **2- 5cm** | 16 | 44 | 10 | 9 | 33 | 17 |
| **>5 cm** | 4 | 7 | 2 | 2 | 7 | 2 |
| **NPI** |  |  |  |  |  |  |
| **>2.4- 3.4** | 2 | 1 | 2 | 1 | 2 | 1 |
| **>3.4- 5.4** | 14 | 47 | 9 | 10 | 27 | 14 |
| **>5.4** | 5 | 8 | 2 | 0 | 13 | 5 |
| **ER** |  |  |  |  |  |  |
| **Negative** | 3 | 10 | 3 | 1 | 12 | 4 |
| **Positive** | 18 | 46 | 10 | 10 | 30 | 16 |
| **PR** |  |  |  |  |  |  |
| **Negative** | 4 | 13 | 4 | 1 | 13 | 5 |
| **Positive** | 17 | 43 | 9 | 10 | 29 | 15 |
| **Her2/neu** |  |  |  |  |  |  |
| **Negative** | 21 | 56 | 13 | 0 | 0 | 0 |
| **Positive** | 0 | 0 | 0 | 11 | 42 | 20 |
| **Metastasis** |  |  |  |  |  |  |
| **Negative** | 20 | 49 | 9 | 8 | 37 | 16 |
| **Positive** | 1 | 7 | 4 | 3 | 5 | 4 |
| **Oper. Type** |  |  |  |  |  |  |
| **Lt MRM** | 12 | 35 | 5 | 6 | 23 | 18 |
| **Rt MRM** | 9 | 21 | 8 | 5 | 19 | 2 |

**Table 4: Distribution of different genotype of INF-γ+874 T/A (rs2430561) gene in different variables of tumor in breast cancer patients (163 Patients) in response to Metastasis in BC group.**

| **Variables** | **AA^-ve^** | **AT^-ve^** | **TT^-ve^** | **AA^+ve^** | **AT^+ve^** | **TT^+ve^** |
| --- | --- | --- | --- | --- | --- | --- |
| **Metastasis** | 29 | 86 | 24 | 4 | 12 | 8 |
| **Cancer stage** |  |  |  |  |  |  |
| **T1** | 3 | 19 | 3 | 0 | 0 | 1 |
| **T2** | 12 | 54 | 18 | 3 | 8 | 5 |
| **T3** | 4 | 9 | 3 | 1 | 3 | 1 |
| **T4** | 0 | 4 | 0 | 0 | 1 | 1 |
| **Node Status** |  |  |  |  |  |  |
| **N0** | 9 | 33 | 9 | 1 | 3 | 3 |
| **N1** | 6 | 21 | 6 | 0 | 6 | 1 |
| **N2** | 9 | 18 | 8 | 1 | 1 | 3 |
| **N3** | 5 | 14 | 1 | 2 | 2 | 1 |
| **Overall grade** |  |  |  |  |  |  |
| **G1** | 0 | 3 | 0 | 0 | 0 | 0 |
| **G2** | 22 | 62 | 19 | 2 | 7 | 4 |
| **G3** | 7 | 21 | 5 | 2 | 5 | 4 |
| **Tumor size** |  |  |  |  |  |  |
| **<2cm** | 2 | 7 | 1 | 0 | 0 | 1 |
| **2- 5cm** | 22 | 68 | 21 | 3 | 9 | 5 |
| **>5 cm** | 5 | 11 | 2 | 1 | 3 | 2 |
| **NPI** |  |  |  |  |  |  |
| **>2.4- 3.4** | 3 | 6 | 0 | 0 | 0 | 0 |
| **>3.4- 5.4** | 19 | 67 | 20 | 2 | 8 | 5 |
| **>5.4** | 7 | 13 | 4 | 2 | 4 | 3 |
| **ER** |  |  |  |  |  |  |
| **Negative** | 4 | 22 | 4 | 0 | 0 | 3 |
| **Positive** | 25 | 64 | 20 | 4 | 12 | 5 |
| **PR** |  |  |  |  |  |  |
| **Negative** | 5 | 22 | 5 | 0 | 4 | 4 |
| **Positive** | 24 | 64 | 19 | 4 | 8 | 4 |
| **Her2/neu** |  |  |  |  |  |  |
| **Negative** | 20 | 48 | 9 | 1 | 7 | 4 |
| **Positive** | 9 | 38 | 15 | 3 | 5 | 4 |
| **Metastasis** |  |  |  |  |  |  |
| **Negative** | 29 | 86 | 24 | 0 | 0 | 0 |
| **Positive** | 0 | 0 | 0 | 4 | 12 | 8 |
| **Oper. Type** |  |  |  |  |  |  |
| **Lt MRM** | 15 | 50 | 17 | 3 | 7 | 5 |
| **Rt MRM** | 14 | 35 | 7 | 1 | 5 | 3 |

**Table 5: Distribution of different genotype of INF-γ+874 T/A (rs2430561) with risk estimate in response to Estrogen receptor (ER) marker in BC group.**

| **Model** | **Genotype # (%)** | | **OR (95% CI)** | **P** |
| --- | --- | --- | --- | --- |
| **Codominant** | **Negative 33 (20.3)** | **Positive 130 (79.7)** |  |  |
| **AA** | 4 (12.1) | 28 (21.5) | 1 |  |
| **AT** | 22 (66.7) | 76 (58.5) | 0.49 (0.15- 1.56) | 0.16 |
| **TT** | 7 (21.2) | 26 (20) | 0.53 (0.14- 2.02) | 0.27 |
| **Dominant** | AA vs AT+ TT | | 0.5 (0.16- 1.55) | 0.16 |
| **Recessive** | AA+ AT vs TT | | 1.1 (0.43- 2.8) | 0.5 |
| **Overdominant** | AA+ TT vs AT | | 1.42 (0.64- 3.17) | 0.25 |

**Table 6: Distribution of different genotype of INF-γ+874 T/A (rs2430561) with risk estimate in response to Progesterone receptor (PR) marker in BC group.**

| **Model** | **Genotype # (%)** | | **OR (95% CI)** | **P** |
| --- | --- | --- | --- | --- |
| **Codominant** | **Negative 40 (24.5)** | **Positive 123 (75.5)** |  |  |
| **AA** | 5 (12.5) | 27 (22) | 1 |  |
| **AT** | 26 (65) | 72 (58.5) | 0.51 (0.18- 1.47) | 0.15 |
| **TT** | 9 (22.5) | 24 (19.5) | 0.49 (0.14- 1.68) | 0.2 |
| **Dominant** | AA vs AT+ TT | | 0.51 (0.18- 1.42) | 0.13 |
| **Recessive** | AA+ AT vs TT | | 1.2 (0.5- 2.85) | 0.42 |
| **Overdominant** | AA+ TT vs AT | | 1.31 (0.62- 2.76) | 0.29 |

**Table 7: Distribution of different genotype of INF-γ+874 T/A (rs2430561) with risk estimate in response to Metastasis status in BC group.**

| **Model** | **Genotype # (%)** | | **OR (95% CI)** | **P** |
| --- | --- | --- | --- | --- |
| **Codominant** | **Negative 24 (14.7)** | **Positive 139 (85.3)** |  |  |
| **AA** | 4 (16.7) | 29 (20.8) | 1 |  |
| **AT** | 12 (50) | 86 (61.8) | 0.99 (0.29- 3.3) | 0.62 |
| **TT** | 8 (33.3) | 24 (17.4) | 0.41 (0.11- 1.54) | 0.15 |
| **Dominant** | AA vs AT+ TT | | 0.76 (0.24- 2.4) | 0.43 |
| **Recessive** | AA+ AT vs TT | | 2.4 (0.92- 6.23) | 0.06 |
| **Overdominant** | AA+ TT vs AT | | 0.61 (0.26- 1.47) | 0.19 |

**Table 8: Distribution of different genotype of INF-γ+874 T/A (rs2430561) with risk estimate in response to Operation Type in BC group.**

| **Model** | **Genotype # (%)** | | **OR (95% CI)** | **P** |
| --- | --- | --- | --- | --- |
| **Codominant** | **Lt MRM 100 (61.4)** | **Rt MRM 63 (38.6)** |  |  |
| **AA** | 18 (18) | 14 (22.2) | 1 |  |
| **AT** | 58 (58) | 40 (63.5) | 0.88 (0.39- 1.98) | 0.46 |
| **TT** | 24 (24) | 9 (14.3) | 0.48 (0.17- 1.36) | 0.13 |
| **Dominant** | AA vs AT+ TT | | 0.77 (0.35- 1.68) | 0.32 |
| **Recessive** | AA+ AT vs TT | | 1.89 (0.81- 4.39) | 0.09 |
| **Overdominant** | AA+ TT vs AT | | 0.79 (0.41- 1.52) | 0.29 |

**Table 9: Distribution of different genotype of INF-γ+874 T/A (rs2430561) with risk estimate in Triple –ve (very poor prognostic model) of hormonal status vs good prognostic hormonal status luminal A model, (ER^+ve^ PR^+ve^ Her2^-ve^) in BC group.**

| **Model** | **Genotype # (%)** | | **OR (95% CI)** | **P** |
| --- | --- | --- | --- | --- |
| **Codominant** | **good prognostic 64 cases** | **prognostic very poor 11 cases** |  |  |
| AA | 17 (26.6) | 3 (27.3) | 1 |  |
| AT | 39 (60.9) | 6 (54.5) | 0.87 (0.19- 3.9) | 0.56 |
| **TT** | 8 (12.5) | 2 (18.2) | 1.41 (0.19- 10.22) | 0.55 |
| **Dominant** | AA vs AT+ TT | | 0.96 (0.23- 4.06) | 0.61 |
| **Recessive** | AA+ AT vs TT | | 0.64 (0.11- 3.52) | 0.45 |
| **Overdominant** | AA+ TT vs AT | | 1.3 (0.36- 4.72) | 0.46 |

**Table 10: Distribution of different genotype of INF-γ+874 T/A (rs2430561) with risk estimate in poor prognostic hormonal status Her2 enriched model (ER^-ve^ PR^-ve^ Her2^+ve^) of vs good prognostic hormonal status luminal A model, (ER^+ve^ PR^+ve^ Her2^–ve^) in BC group.**

| **Model** | **Genotype # (%)** | | **OR (95% CI)** | **P** |
| --- | --- | --- | --- | --- |
| **Codominant** | **good prognostic 64 cases** | **poor prognostic 14cases** |  |  |
| AA | 17 (26.6) | 1 (7.1) | 1 |  |
| AT | 39 (60.9) | 10 (71.4) | 4.36 (0. 51- 36.79) | 0.13 |
| **TT** | 8 (12.5) | 3 (21.5) | 6.37 (0.57- 71.27) | 0.13 |
| **Dominant** | AA vs AT+ TT | | 4.7 (0.57- 38.7) | 0.10 |
| **Recessive** | AA+ AT vs TT | | 0.52 (0.12- 2.29) | 0.31 |
| **Overdominant** | AA+ TT vs AT | | 0.62 (0.17- 2.21) | 0.33 |

**Table 11: Distribution of different genotype of INF-γ+874 T/A (rs2430561) with risk estimate in Triple –ve (very poor prognostic model) of hormonal status vs poor prognostic hormonal status Her2 enriched model (ER^-ve^ PR^-ve^ Her2^+ve^) in BC group.**

| **Model** | **Genotype # (%)** | | **OR (95% CI)** | **P** |
| --- | --- | --- | --- | --- |
| **Codominant** | **prognostic very poor 11 cases** | **poor prognostic 14 cases** |  |  |
| AA | 3 (27.3) | 1 (7.1) | 1 |  |
| AT | 6 (54.5) | 10 (71.4) | 5.0 (0. 42- 59.65) | 0.21 |
| **TT** | 2 (18.2) | 3 (21.5) | 4.5 (0.25- 80.56) | 0.35 |
| **Dominant** | AA vs AT+ TT | | 4.87 (0.43- 55.29) | 0.20 |
| **Recessive** | AA+ AT vs TT | | 0.81 (0.11- 5.98) | 0.62 |
| **Overdominant** | AA+ TT vs AT | | 0.48 (0.09- 2.52) | 0.32 |

**Table 12: Means and standard error of the mean of NPI for different genotype of INF-γ+874 T/A (rs2430561) in BC group.**

| **IFN** | | **N** | **Mean** | **Std. Deviation** | **Std. Error** | **Sig.^a^** |
| --- | --- | --- | --- | --- | --- | --- |
| **NPI** | **AA** | 32 | 4.826 | 0.8644 | 0.1528 |  |
|  | **AT** | 98 | 4.653 | 0.7888 | 0.0797 | 0.297 |
|  | **TT** | 33 | 4.691 | 0.7935 | 0.1381 | 0.515  0.297**^b^** |

**a= significance of AA genotype vs other genotype., b= significance of AT genotype vs TT genotype.**

**Table 13: Means and standard error of the mean of NPI for different hormonal marker status in BC group.**

| **NPI** | | **N** | **Mean** | **Std. Deviation** | **Std. Error** | **Sig.** |
| --- | --- | --- | --- | --- | --- | --- |
| **ER** | **negative** | 33 | 4.618 | 0.8400 | 0.1462 |  |
|  | **positive** | 130 | 4.714 | 0.7951 | 0.0697 | 0.537 |
| **PR** | **negative** | 38 | 4.792 | 0.8882 | 0.1441 |  |
|  | **positive** | 125 | 4.665 | 0.7762 | 0.0694 | 0.395 |
| **HER2neu** | **negative** | 88 | 4.592 | 0.7765 | 0.0828 |  |
|  | **positive** | 75 | 4.816 | 0.8211 | 0.0948 | 0.076 |

**Table 14: Means and standard error of the mean of NPI for different genotype of INF-γ+874 T/A (rs2430561) in response to Estrogen receptor (ER) expression marker in BC group.**

| **IFN** | | **ER** | **N** | **Mean** | **Std. Error** | **Sig** |
| --- | --- | --- | --- | --- | --- | --- |
| **AA** |  | **negative** | 4 | 4.720 | 0.5001 |  |
|  |  | **positive** | 28 | 4.841 | 0.1631 | 0.799 |
| **AT** |  | **negative** | 22 | 4.514 | 0.1611 |  |
|  |  | **positive** | 76 | 4.694 | 0.0916 | 0.348 |
| **TT** |  | **negative** | 7 | 4.886 | 0.4026 |  |
|  |  | **positive** | 26 | 4.638 | 0.1414 | 0.473 |

**Table 15: Means and standard error of the mean of NPI for different genotype of INF-γ+874 T/A (rs2430561) in response to Progesterone receptor (PR) expression marker in BC group.**

| **IFN** | | **PR** | **N** | **Mean** | **Std. Error** | **Sig** |
| --- | --- | --- | --- | --- | --- | --- |
| **AA** |  | **negative** | 3 | 5.167 | 0.3180 |  |
|  |  | **positive** | 29 | 4.790 | 0.1652 | 0.366 |
| **AT** |  | **negative** | 26 | 4.715 | 0.1699 |  |
|  |  | **positive** | 72 | 4.631 | 0.0900 | 0.643 |
| **TT** |  | **negative** | 9 | 4.889 | 0.3565 |  |
|  |  | **positive** | 24 | 4.617 | 0.1380 | 0.389 |

**Table 16: Means and standard error of the mean of NPI for different genotype of INF-γ+874 T/A (rs2430561) in response to Her2/neu expression marker in BC group.**

| **IFN** | | **HER2neu** | **N** | **Mean** | **Std. Error** | **Sig** |
| --- | --- | --- | --- | --- | --- | --- |
| **AA** |  | **negative** | 19 | 4.778 | 0.1616 |  |
|  |  | **positive** | 13 | 4.895 | 0.3009 | 0.712 |
| **AT** |  | **negative** | 56 | 4.516 | 0.1034 |  |
|  |  | **positive** | 42 | 4.837 | 0.1204 | 0.046 |
| **TT** |  | **negative** | 13 | 4.646 | 0.2480 |  |
|  |  | **positive** | 20 | 4.720 | 0.1663 | 0.799 |
